# Supplementary material for: Targeting Trypanothione Reductase, a Key Enzyme in the Redox Trypanosomatid Metabolism, to Develop New Drugs against Leishmaniasis and Trypanosomiases
Source: Molecules. 2020 Apr 21;25(8):1924. doi: 10.3390/molecules25081924 (PMC7221613; doi:10.3390/molecules25081924)
Supplement: Supplementary file 1 [file molecules-25-01924-s001.pdf]

Review

Targeting Trypanothione Reductase, a Key Enzyme in the Redox Trypanosomatid Metabolism, to Develop New Drugs against Leishmaniasis and Trypanosomiases

Theo Battista [1], Gianni Colotti [2], Andrea Ilari [2] and Annarita Fiorillo [1,*]

[1] Department of Biochemical Sciences, Sapienza University, P.le A.Moro 5, 00185, Rome, Italy; theo.battista@uniroma1.it

[2] Institute of Molecular Biology and Pathology, Italian National Research Council, IBPM-CNR, c/o Department of Biochemical Sciences, Sapienza University, P.le A.Moro 5, 00185, Rome, Italy; gianni.colotti@cnr.it (G.C.); andrea.ilari@cnr.it (A.I.)

* Correspondence: annarita.fiorillo@uniroma1.it; Tel.: +39-064-991-0910

Table S1. IUPAC name and smiles of inhibitors.

PDB ID	Ligand PDB ID	iupac name	smiles	structure
4NEV	2JR	5-{5-[1-(pyrrolidin-1-yl)cyclohexyl]-1,3-thiazol-2-yl}-1*H*-indole	c1cc2c(cc[nH]2)cc1c3ncc(s3)C4(CCCCC4)N5CCCC5	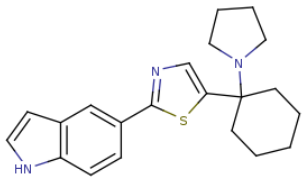
4NEW	2JR	5-{5-[1-(pyrrolidin-1-yl)cyclohexyl]-1,3-thiazol-2-yl}-1*H*-indole	c1cc2c(cc[nH]2)cc1c3ncc(s3)C4(CCCCC4)N5CCCC5	
6ER5	BVN	2-(diethylamino)ethyl 4-((3-(4-nitrophenyl)-3-oxopropyl)amino)benzoate	CCN(CC)CCOC(=O)c1ccc(cc1)NCCC(=O)c2ccc(cc2)[N+](=O)[O-]	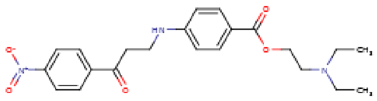
6OEZ	M9J	*N*-(cyclobutylmethyl)-3-{5-[1-(pyrrolidin-1-yl)cyclohexyl]-2-(1-{[(2S)-pyrrolidin-2-yl]methyl}-1*H*-indol-5-yl)-1,3-thiazol-4-yl}prop-2-yn-1-amine	c1cc2c(ccn2C[C@@H]3CCCN3)cc1c4nc(c(s4)C5(CCCCC5)N6CCCC6)C#CCNCC7CCC7	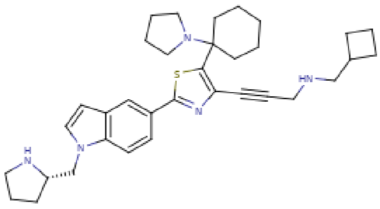

6OEY	M9S	5-{5-[1-(pyrrolidin-1-yl)cyclohexyl]-1,3-thiazol-2-yl}-1-{[(2S)-pyrrolidin-2-yl]methyl}-1*H*-indole	c1cc2c(ccn2C[C@@H]3CCCN3)cc1c4ncc(s4)C5(CCCCC5)N6CCCC6	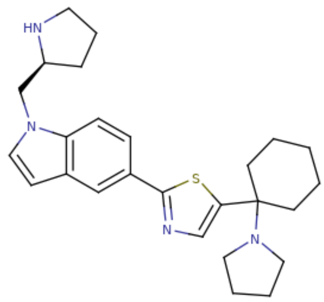
60EX	M9Y	3-(2-{1-[2-(piperidin-4-yl)ethyl]-1*H*-indol-5-yl}-5-[1-(pyrrolidin-1-yl)cyclohexyl]-1,3-thiazol-4-yl)-*N*-(2,2,2-trifluoroethyl)prop-2-yn-1-amine	c1cc2c(ccn2CCC3CCNCC3)cc1c4nc(c(s4)C5(CCCCC5)N6CCCC6)C#CCNCC(F)(F)F	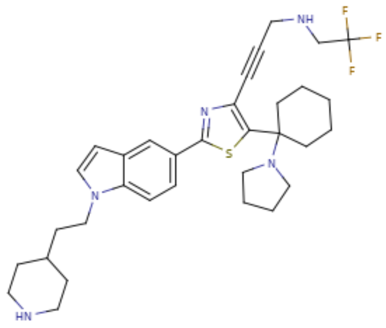
1GXF	QUM	(4R)-*N'*-(6-chloro-2-methoxy-acridin-9-yl)-*N,N*-diethyl-pentane-1,4-diamine	C[C@@H](CCCN(CCCl)CCCl)/N=C\1/c2ccc(cc2Nc3c1cc(cc3)OC)Cl	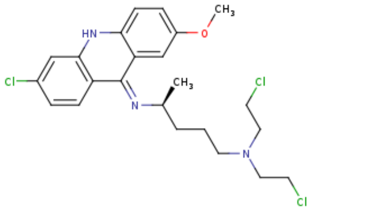
6BU7	RD0	1-[2-(piperidin-4-yl)ethyl]-5-{5-[1-(pyrrolidin-1-yl)cyclohexyl]-1,3-thiazol-2-yl}-1*H*-indole	c1cc2c(ccn2CCC3CCNCC3)cc1c4ncc(s4)C5(CCCCC5)N6CCCC6	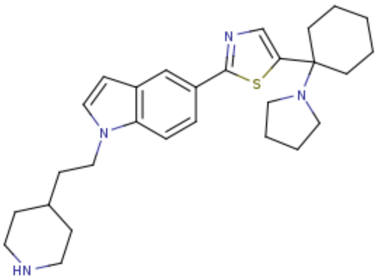
6BTL	RD7	1-[2-(piperazin-1-yl)ethyl]-5-{5-[1-(pyrrolidin-1-yl)cyclohexyl]-1,3-thiazol-2-yl}-1*H*-indole	c1cc2c(ccn2CCN3CCNCC3)cc1c4ncc(s4)C5(CCCCC5)N6CCCC6	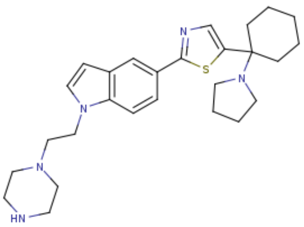
5EBK	RDS	6-sec-Butoxy-2-[(3-chlorophenyl)sulfanyl]-4-pyrimidinamine	CC[C@@H](C)Oc1cc(nc(n1)Sc2cccc(c2)Cl)N	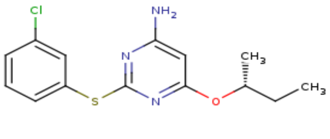

2YAU	TS8	3,4,5-triacetyloxy-6-(acetyloxymethyl)oxane-2-thiol	CC(=O)OC[C@@H]1[C@H]([C@@H]([C@H]([C@@H](O1)S)OC(=O)C)OC(=O)C)OC(=O)C	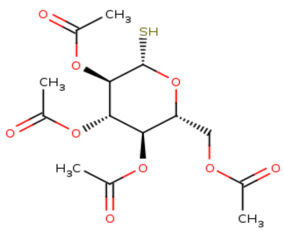
2WP5	WP5	methyl [(4S)-6-bromo-2-methyl-4-phenylquinazolin-3(4*H*)-yl]acetate	CC1=Nc2ccc(cc2[C@@H](N1CC(=O)OC)c3ccccc3)Br	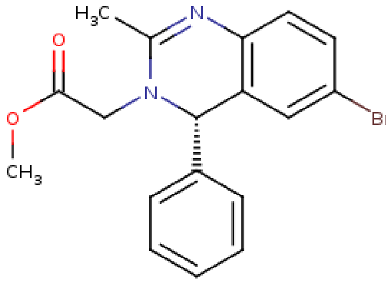
2WP6	WP6	(4S)-3-benzyl-6-chloro-2-methyl-4-phenyl-3,4-dihydroquinazoline	CC1=Nc2ccc(cc2[C@@H](N1Cc3ccccc3)c4ccccc4)Cl	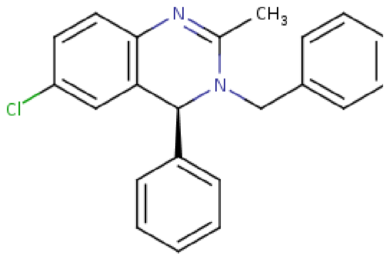
2WPC	WP7	(4S)-6-chloro-3-{2-[4-(furan-2-ylcarbonyl)piperazin-1-yl]ethyl}-2-methyl-4-phenyl-3,4-dihydroquinazoline	CC1=Nc2ccc(cc2[C@@H](N1CCN3CCN(CC3)C(=O)c4ccco4)c5ccccc5)Cl	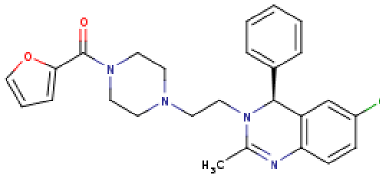
2WPE	WPE	*N*-{2-[(4S)-6-chloro-2-methyl-4-phenylquinazolin-3(4h)-yl]ethyl}furan-2-carboxamide	CC1=Nc2ccc(cc2[C@@H](N1CCNC(=O)c3ccco3)c4ccccc4)Cl	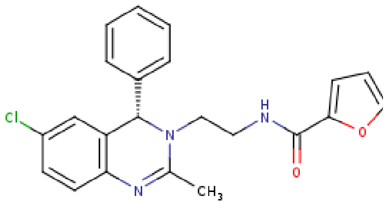
2WPF	WPF	3-[(4S)-6-chloro-2-methyl-4-(4-methylphenyl)quinazolin-3(4h)-yl]-n,n-dimethylpropan-1-amine	Cc1ccc(cc1)[C@H]2c3cc(ccc3N=C(N2CCCN(C)C)C)Cl	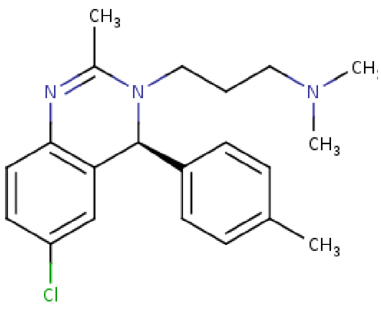

4APN (B)	JV0	4-[[1-(4-ethylphenyl)-2-methyl-5-(4-methylsulfanylphenyl)pyrrol-3-yl]methyl]thiomorpholine	CCc1ccc(cc1)-n1c(C)c(CN2CCSCC2)cc1-c1ccc(SC)cc1	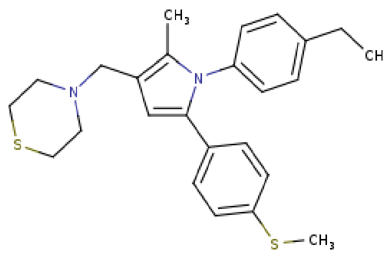
6I7N (B)	H6H	~{N}-(4-azanylbutyl)-~{N}-(2-azanyl-2-oxidanylidene-ethyl)-7-(3-azanyl-3-oxidanylidene-propyl)-4-(dimethylamino)-2-(2-naphthalen-2-ylethylamino)pyrrolo[2,3-d]pyrimidine-6-carboxamide	CN(C)c1nc(NCCc2ccc3ccccc3c2)nc2n(CCC(N)=O)c(cc12)C(=O)N(CCCCN)CC(N)=O	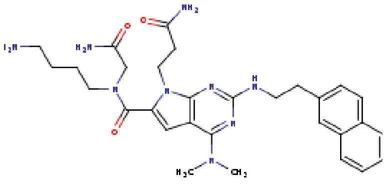
	JWZ	rac-4-(((3-(8-(2-((1*R*,2*S*,5*R*)-6,6-dimethylbicyclo[3.1.1]heptan-2-yl)ethyl)-4-oxo-1-phenyl-1,3,8-triazaspiro[4.5]decan-3-yl)propyl)(methyl)amino)methyl)-4-hydroxypiperidine-1-carboximidamide	N=C(N)N1CCC(O)(CC1)CN(C)CCCN6CN(c2ccccc2)C3(CCN(CC3)CC[C@@H]5CC[C@H]4C[C@@H]5C4(C)C)C6=O	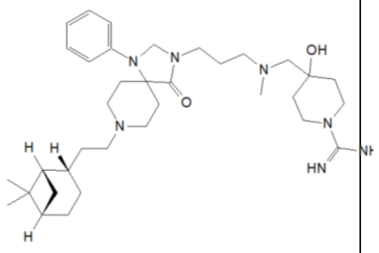
